# Psychosocial interventions reduce cortisol in breast cancer patients: systematic review and meta-analysis

**DOI:** 10.3389/fpsyg.2023.1148805

**Published:** 2023-06-27

**Authors:** Edith Mészáros Crow, Rosa López-Gigosos, Eloisa Mariscal-López, Marina Agredano-Sanchez, Natalia García-Casares, Alberto Mariscal, Mario Gutiérrez-Bedmar

**Affiliations:** ^1^Department of Public Health and Psychiatry, School of Medicine, University of Málaga, Málaga, Spain; ^2^Biomedical Research Institute of Málaga-IBIMA, Málaga, Spain; ^3^Department of Medicine, School of Medicine, University of Málaga, Málaga, Spain; ^4^Centro de Investigaciones Medico-Sanitarias (C.I.M.E.S), Málaga, Spain; ^5^CIBERCV Cardiovascular Diseases, Carlos III Health Institute, Madrid, Spain

**Keywords:** psychosocial intervention, cortisol, breast cancer, recurrence, meta analysis

## Abstract

**Introduction:**

Cancer initiation, progression and recurrence are intricate mechanisms that depend on various components: genetic, psychophysiological, or environmental. Exposure to chronic stress includes fear of recurrence that can affect biological processes that regulate immune and endocrine systems, increase cancer risk, and influence the survival rate. Previous studies show that psychological interventions might influence the level of cortisol that has been extensively used as a biomarker for measuring hypothalamic-pituitary-adrenal axis functioning and body's immunity response. This meta-analysis aimed to provide a quantitative scrutiny of the effect of certain types of psychosocial interventions on cortisol as a neuroendocrine biomarker in saliva or blood and might predict breast cancer (BC) progression.

**Methods:**

A literature search was performed in the following databases: PubMed, The Cohrane Library, Scopus, WOS, PsychInfo, Google Scholar, Ovid Science Direct. After methodical selection of originally generated 2.021 studies, the search yielded eight articles that met inclusion criteria. All these studies explored effects of psychosocial interventions that measured cortisol in total of 366 participants with BC, stages 0-IV, in randomized control trial or quasi experimental study design setting. We applied random effects model to conduct meta-analyses on the parameters of salivary and plasma cortisol and used PRISMA Guidelines as validated methodology of investigation to report the results.

**Results:**

Eight studies selected for meta-analysis have shown the reduction of cortisol level due to applied psychosocial intervention. The random effects model showed that interventions produced large effect sizes in reductions of cortisol in blood (Cohen's d = −1.82, 95% Confidence Interval (CI): −3.03, −0.60) and slightly less in saliva (d = −1.73, 95%CI: −2.68, −0.78) with an overall effect of d = −1.76 (95%CI: −2.46, −1.07).

**Conclusion:**

Our study concluded that certain types of psychosocial interventions reduce cortisol (indicator of chronic stress) in patients with BC. Application of specific psychosocial support as adjuvant non-invasive therapy for affected females with BC at all phases of treatment could contribute to more cost-effective health care.

## Introduction

Cancer initiation, progression, recurrence, and metastasis are intricate mechanisms that depend on various components. Those components include genetic alterations, proliferation, vascularization, invasion, embolization, and evasion of apoptosis. When a tumor is established, its growth, metastatic spread, or eventual recovery, heavily depend on interactions with the microenvironment and psychological state of a patient (Armaiz-Pena et al., 2009). Despite the prevalence of cancer recurrence, research of primary psychological causes that might trigger the disease relapse are limited. Stress and fear of recurrence can also negatively affect biological processes that regulate immune and endocrine systems, influence the survival rate and the quality of life. Approximately 7% of patients develop disabling emotional condition including intrusive thoughts and misinterpretation of mild and unrelated symptoms. Patients with breast cancer (BC) have up to 30% of recurrence rate, and the timing of relapse varies considerably, influenced by classic prognostic factors (the axillary lymph node status, the tumor size, and the nuclear grade and histological grade) as well as the choice of adjuvant treatment strategies (Colleoni et al., 2016).

The new promising microbiological development proposes to suppress cancer relapse and metastasis by inhibiting cancer stem cells (Li et al., 2015). Not less important or complex research line exploring tumor development is related to the study of behavioral stress and immune or neuroendocrine biomarkers' changeability in response to stress. Psychoneuroimmunology (PNI) investigates in detail the connection between the psychological stress, its impact on neuro-immune system and cancer incidence or progression. Psychological stress and adverse life events can impact cellular immune response dysregulating homeostatic functionality on a cellular level and harm the protective functions of the immune system (Glaser and Kiecolt-Glaser, 2005). Specific signaling pathways are activated through neuroendocrine stress response resulting in promotion of tumor growth and metastasis. Neuroendocrine and immunological mediators of stress impact sympathetic nervous system and diverse interaction has been identified between malignant tissues and immune-cites (Batty et al., 2017). Oncologists now implicate psychological functioning in the prediction of cancer outcomes.

Finding a “gold standard” biomarker to measure body's response to allostatic load or experiential input (social, emotional, and physical experiences) has been proven to be challenging, given its complex etiology and highly individual manifestations. Cortisol, synthesized from cholesterol and released into the body fluids after activating hypothalamic-pituitary-adrenal (HPA) axis, is the main glucocorticoid hormone of the adrenal cortex that can be used as a peripheral indicator of hypothalamic neural activity (Dubey and Boujoukos, 2005) in the process of physiological response to endogenous and exogenous stressors. Evidence of recent research in molecular biology shows relationship between dysregulation of HPA axis, abnormal secretion of cortisol and protumorigenic pathways within breast cells (Figueira et al., 2022). When the HPA axis is chronically activated due to stress events and fears (major contributors being negative lifestyle, fast paced jobs, existential fears, negative emotional experiences, anxiety, depression), cortisol levels rise, and inflammatory responses are downregulated. As a response to biochemical stress, its secretion contributes to the suppression of the “stress axis” on mental health, where more inflammatory ligands (cytokines) are released into circulation in the vicinity of tumor cells (Levine et al., 2007; Lee et al., 2015). This proliferation of cytokines may affect disease progression by causing angiogenesis and endothelial-to-mesenchymal transition (EndMT) leading to metastasis (Morris et al., 2022).

Some molecular studies also suggest that cortisol's immunosuppressive effect may result in inability to combat tumor formation, reduced immunosurveillance of early-stage cancer, facilitating the immune escape and acquisition of further oncogenic mutations (Antonova et al., 2011; Coutinho and Chapman, 2011; Al Sorkhy et al., 2018). Additionally, cortisol has hyperglycaemic and obesogenic effects where both dysregulations lead to increased risk of malignancies (Nead et al., 2015). More specifically, stimulation of BC development by prolonged or elevated presence of cortisol during periods of stress may also partially occur as a result of augmented estrogen biosynthesis (Aromatase activity) (O'Neill et al., 1988). Thus, both clinical and molecular research reveals a positive correlation of high cortisol levels and the progression of BC, resulting in worse prognosis.

A small fraction of unbound free cortisol is demonstrated to be biologically active, coming out of the mitochondrion, whereas in general cortisol binds to cytosolic receptors. Cortisol migrates out of the cell into the extracellular space and into the bloodstream. Due to its low molecular weight and lipophilic nature, unbound cortisol enters the cells through passive diffusion, which makes it feasible to measure free cortisol in many body fluids. Several studies explored the potential of using cortisol as an indicator to assess cumulative biological risks, predict chronic stress, reduce the incidence of pre-phase of chronic illnesses (Dubey and Boujoukos, 2005; Levine et al., 2007; Lee et al., 2015).

Among its various manifestations, cortisol parameters are compounded of both serum (blood) and salivary measures. Levels of circulating cortisol reflect the activity of central and peripheral pathways that are responsive to experiential input. It has also been observed that 70% of BC patients show high levels of cortisol (De Brabander and Gerits, 1999). Plasma and salivary cortisol levels rise due to circadian influences as well as perturbations by stressors of the organism's environment that makes it possible to detect rather robust experimental effects (Levine et al., 2007; Lee et al., 2015).

Clinical studies dealing with the relationship between behavioral tendencies and the progression of a disease in patients that were already diagnosed with cancer have found that factors such as lack of social support, negative emotions and their suppression, fear of recurrence, hopelessness and denial are associated with poorer survival (Batty et al., 2017). Several systematic reviews and meta-analysis dedicated to causal relationship of stress, depression with incidence or progression of tumor have been inconclusive or showing only borderline association (De Brabander and Gerits, 1999; Petticrew et al., 1999; Antoni et al., 2006; Batty et al., 2017). Other studies have confirmed the adverse effect of this relationship on cancer incidence and survival (Duijts et al., 2003; Chida et al., 2008; Santos et al., 2009). Finally, in the study of Satin et al. (2009), depression in cancer patients has been exposed to be a predictive factor of mortality but not progression. These clinical data parallel results from animal-based studies, demonstrating that experimentally imposed stress can modulate cancer progression (Mustafa et al., 2013).

When determining the predictive power of psychosocial factors for the course of BC, additional biological factors and markers suggesting progression including tumor-free interval (TFI), site of recurrence, number of metastases, menopausal status and use of different medications should be taken into consideration (Levy et al., 1988; Carter et al., 1989; Lin et al., 2013; Mustafa et al., 2013).

BC is regarded to be one of the most appropriate cancers for studying psychosocial influences on disease progression due to its relatively variable course, high prevalence, and hormone sensitivity (Thaker and Sood, 2008).

Thus, data that would illustrate a relationship between behavioral risk factors and cancer initiation and progression have been inconclusive. To differentiate our research and bring novelty to the subject, this review and meta-analysis is aimed to consolidate existing data up to date (2022) and test the hypothesis if psychosocial interventions (and their variations that are subject of this study) can reduce neuroendocrine biomarker (cortisol) and can be considered as a plausible adjuvant therapy to attenuate chronic stress. The objective of this study is to provide a summary of the studies where primary or secondary outcome measure was cortisol in the population of females with BC in various stages of disease progression. Further, to present an overview of methodological and reporting aspects of these studies and to execute a quantitative analysis of association between the applied intervention and cortisol levels. We focused solely on BC and narrowed the scope of psychosocial interventions to be structured and methodical. We also considered other potential moderators, including characteristics of samples and trial quality by pooling relevant data from studies that met eligibility criteria. Although this review has focused on investigating Changes in the variable of cortisol under the influence of applied psychosocial intervention, neuroendocrine and immunological mechanisms that modulate cancer growth and might prevent cancer progression, metastasis or recurrence will remain outside of the scope of this review.

To our knowledge no other quantitative study devoted to this narrow scope of interventions and outcome measures has been conducted so far.

## Materials and methods

We used the checklist of Preferred Reporting Items for Systematic Reviews and Meta-Analyses (PRISMA) Guidelines as a validated methodology of investigation (Page et al., 2021) to perform the selection procedure, study identification, synthesis methods and critical appraisal.

### Search strategy

Databases of Pubmed, Medline, Web of Science, Scopus, PsychInfo, Embase, Dare, Cinahl (EBSCO), Google Scholar, Cochrane Library, OVID, Science Direct, ProQuest were systematically searched electronically and manually using appropriate controlled vocabulary terms according to Boolean operators' method specific for each database.

The search strategy consisted of the list of key words and Medical Subject Headings (MeSH) terms based on the principal concepts of our research. We applied the following terms: (1) Cognitive Behavioral Therapy, Mindfulness Based Stress Reduction, Psychosocial Therapy (“cognitive behavioral therapy” OR “psych^*^” OR “psychotherapy” OR “psychosocial” OR “psycho-education” OR “mindfulness^*^” OR “mindfulness-based”^*^); (2) breast cancer (“cancer^*^” OR “breast tumor” OR “breast carcinoma” OR “breast neoplasm” OR “mammary carcinoma”); (3) immune and neuroendocrine biomarkers (“biological markers” OR “neuroendocrine biochemical mediators” OR “immune biomarkers” OR “cortisol”). Further restrictions and search formulas were used to narrow the search that included academic publications in English from each database's inception through December 2022. We also examined bibliographical references of the articles identified through the search including relevant reviews or meta-analyses. These were further scrutinized to check whether they contained any other studies suitable for our review.

### Eligibility criteria

Studies were included where the main PICOD (population, intervention, comparison, outcomes, design) concepts were defined as follows:

1. Population: women diagnosed with BC, stages 0-IV according to Tumor, Node, Metastasis (TNM) scale and post-operative tumor free stage (TFI).
2. Interventions: group or individual psychosocial interventions containing cognitive behavioral therapy, cognitive behavioral stress management, mindfulness-based intervention either in isolated form or combined with other modality, psychosocial support and therapy, psychotherapy, mindfulness-based stress reduction, existential cognitive group therapy, hypnotic guided imagery program, supportive expressive group therapy.
3. Control group of usual care or waitlisted comparison.
4. Variable of outcome measures: neuroendocrine biochemical mediator – cortisol.
5. Study design: randomized and non-randomized clinical trials, pre-post measurements. We limited the analysis to controlled or quasi experimental intervention studies.

Studies were excluded if:

1. The studied sample population was not diagnosed with BC. Studies where survivorship was the only outcome without measuring neuroendocrine biomarkers as a primary or secondary outcome.
2. Studies where intervention did not involve psychosocial support (in any of its variations that are subject of this study) in a group or individual format, and only involved exercise, tai chi, qi gong, yoga, aerobic or other modality, or where the intervention narrowed to only verbal, written or telephone self-expression or self-help without any structured program.
3. Studies where the articles did not provide sufficient data to calculate effect sizes or where measurements of cortisol were not explicitly presented in mean and standard deviation (SD) numbers, or if studies had overlapping information.

### Data extraction and synthesis

We prepared beforehand a coding manual to independently mark selected trials according to the inclusion criteria. Two authors (EC and MG-B) have extracted data according to this checklist including various categories related to the following characteristics: (1) study: first author's name, year of publication, country of the study; (2) study subjects: total number of participants, intervention and control group number of persons, mean age, gender, ethnicity, BC stage, recurrence or metastasis if occurred; (3) intervention: intervention design, random or non-random allocation, blinding of investigators, blinding of subjects, sample size, intervention and control type description, group or individual, duration and frequency of treatment, timing of intervention after resection surgery, patient involvement, post-surgery or post-treatment timing, follow up after intervention; (4) outcome variables: measuring techniques, continuous data from assessment of immune or neuroendocrine variables (primarily salivary and plasma cortisol), applied psychological variables, withdrawals, preintervention check of equality of the groups, differences found or not found in target population prior to the intervention, to be included or no in the final list. The manual was revised for discrepancies during the coding to incorporate important aspects of the located studies. We also extracted quantitative data to calculate effect sizes. After the process of selection of studies to be included in meta-analysis, a final list of variables was produced. This list covered baseline cortisol measurements (mean and SD) and end point cortisol measurements (mean and SD) for both intervention and control groups. Where there were multiple assessment time points, we chose post-intervention data collection. We also assessed methodological quality of included studies.

### Risk of bias assessment

The quality of studies was performed by two authors (EC and MB) using methodology tool RoB2 of Cochrane Risk of Bias assessment (Higgins et al., 2022). The studies were labeled according to the outcome measure: salivary or serum cortisol. Assessment was executed for all five domains: (1) risk of bias arising from randomization process, (2) effect of assignment to intervention, (3) missing outcome data, (4) measurements of the outcome variables, (5) selection of the reported results and overall risk of bias. The studies were marked to have low risk, high risk of bias or to have some concerns (where the information was not available or unclear). The consensus of both evaluators was reached unanimously.

### Statistical considerations

To evaluate the change in cortisol levels produced by the psychosocial intervention in each study, the mean difference in cortisol levels at baseline and at the end of the intervention and the SD of this difference were calculated. In the control group of each study the mean differences and SDs were also evaluated. To estimate the effect of each intervention on changes in cortisol levels vs. the control group, we used Cohen's d (d) and its 95% confidence interval (CI) as a measure of effect size. A |d| value of 0.80 or higher was considered to represent a large effect size. These effects were considered as statistically significant if the CI did not include zero.

To assess heterogeneity across studies we examined forest plots and used Cochrane's Q test and I^2^ statistics. I^2^ > 75% was considered to present high degree of heterogeneity. In the cases of high heterogeneity, random effect models (DerSimonian and Laird method) were used. Potential publication bias was assessed through visual inspection of funnel plot and by Egger's and Begg's tests.

A p-curve analysis for a set of reported data was performed to observe distribution of statistically significant *p*-values (*p* ≤ 0.05). All statistical analyses were performed with Stata version 17.0 (Stata Corp). All statistical tests were two-sided, *i* and p-value < 0.05 was considered statistically significant.

## Results

### Search process

A preliminary search of eight databases identified 2.021 studies [(Pubmed/Medline (*n* = 507), Scopus (*n* = 156), WOS (*n* = 33), PsychInfo (*n* = 17), Google Scholar (*n* = 696), Cochrane (*n* = 389), Ovid (*n* = 192), Science Direct (*n* = 31)]. We have reached the search results of 417 articles through relevant filtering of each database, preliminary screening of the article titles, and identifying additional 27 records through other sources (examining references of certain narrative reviews). We also extracted any duplicates. Upon abstract screening, we removed 274 articles, thus reviewing full text of 143 articles. We further excluded 77 studies because they were not intervention studies (but narrative or systematic reviews) and only focused on 69 records presenting experimental or observational studies.

As a result, 36 articles met inclusion criteria and were selected for the preliminary qualitative analysis (that explored effects of psychosocial interventions with neuroendocrine and immune mediators as primary or secondary outcome). Other articles were excluded because they measured primarily survival or other parameters as an outcome of intervention effect which is outside of the scope of this research. We further classified these articles into three larger groups according to the immune or neuroendocrine biomarkers as an outcome: cortisol, lymphocytes, and pro-inflammatory cytokines in order not to lose the cortisol values if cortisol was not the primary or sole outcome of the study. Since the current paper is based on cortisol (both salivary and plasma), 16 reports were pre-selected into this category (Davis, 1986; Gruber et al., 1993; Schedlowski, 1994; van der Pompe et al., 1997; Cruess et al., 2000; Chan et al., 2006; Nunes et al., 2007; Witek-Janusek et al., 2008; Antoni et al., 2009; Matchim et al., 2011; Phillips et al., 2011; Baker et al., 2012; Hsiao et al., 2012; Dodds et al., 2015; Webster et al., 2016; Lengacher et al., 2019). When published articles did not present sufficient data to calculate the effect size, we contacted authors for the required information. However, for the remaining seven articles, the authors either did not respond or were unable to provide essential data (Davis, 1986; Gruber et al., 1993; van der Pompe et al., 1997; Nunes et al., 2007; Witek-Janusek et al., 2008; Antoni et al., 2009; Webster et al., 2016; Lengacher et al., 2019), except one study of Baker et al. (2012), where we obtained additional information.

**Figure 1:**
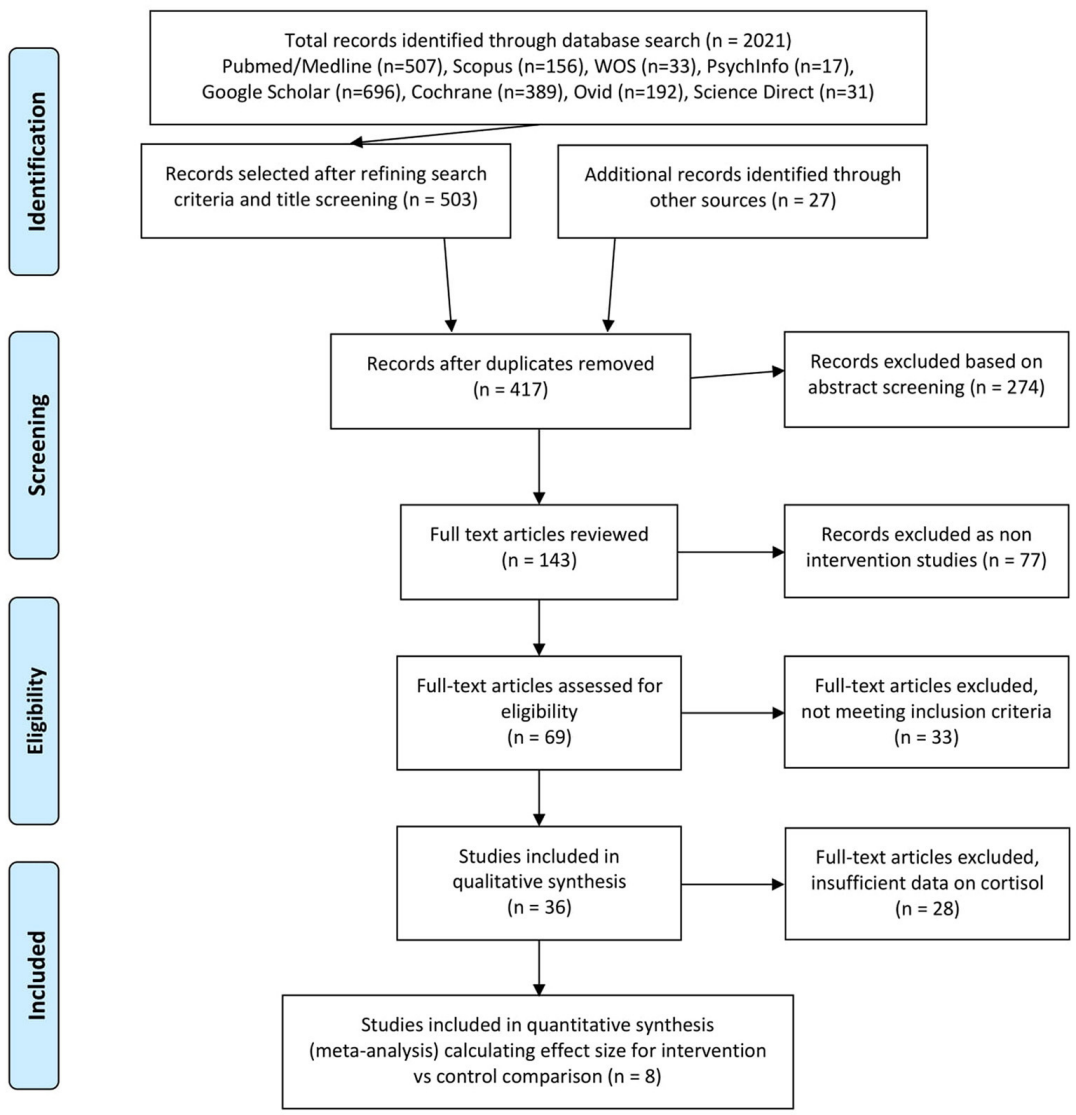
Literature selection PRISMA flow chart.

Therefore, only eight studies exhibited sufficient data to calculate effect size and additional data that included pre- and all postintervention means, SDs, and sample sizes for each treatment arm. Thus, eight unique trials (Schedlowski, 1994; Cruess et al., 2000; Chan et al., 2006; Matchim et al., 2011; Phillips et al., 2011; Baker et al., 2012; Hsiao et al., 2012; Dodds et al., 2015) that included a total of 366 participants with BC types (stages 0-IV) who had been randomly and non-randomly assigned to treatment groups were included in the meta-analysis for effect size computing and calculation of intervention vs. control data comparison. The flow chart of our search strategy according to PRISMA methodology is presented in Figure 1.

### Study characteristics

All included articles aimed to compare psychophysiological effects of different psychosocial interventions vs. no intervention (control group) or waiting list group in BC patients. Four studies were executed in the US (Cruess et al., 2000; Matchim et al., 2011; Phillips et al., 2011; Dodds et al., 2015), two studies originated from Hong Kong, China (Chan et al., 2006; Hsiao et al., 2012), one from Germany (Schedlowski, 1994) and one from UK (Baker et al., 2012). A total sample (*N* = 366) of patients across all eight studies who were diagnosed with primary BC (stages 0-IV), with mean age ranging from 45.6 to 61.5 was included in our research analysis. Subjects were undergoing chemotherapy, radiation therapy and/or hormone therapy or were prior to proceeding to adjuvant therapy process. The population was rather homogeneous, no significant difference in demographic, disease and medical treatment variables were found at baseline.

Interventions were: (1) Haven integrated support program including psychological support, information, and a range of complementary therapies specifically designed to support the physical and emotional needs of patients with BC before, during and after standard medical treatment (therapy consultation, nutrition, counseling, touch therapies, herbal medicine, homeopathy, acupuncture, yoga, meditation, Qigong) (Baker et al., 2012); (2) Body Mind Spirit (BMS) integrated group therapy based on Chan's “Cancer Fighter” training manual consisting of concepts of positive psychology and forgiveness therapy, social support, purpose of meaning of life, developing equability in the mind and body, positive psychology on self-acceptance, autonomy, environmental mastery, personal growth, a sense of purpose in life (Hsiao et al., 2012); (3) Meditation-based program called Cognitively-Based Compassion Training (CBCT) consisting of meditative concentration, non-judgmental awareness of thoughts, self-compassion, stress reactivity, appreciation and gratitude, empathy and compassion practices, aspiration for happiness (Dodds et al., 2015); (4) Cognitive-Behavioral Stress Management (CBSM) consisting of stress management techniques (cognitive restructuring, coping skills training, assertiveness, anger management, and social support utilization skills) and relaxation training components (e.g., progressive muscle relaxation, meditation, abdominal breathing, and guided imagery) – same structured intervention applied in studies of Cruess (Cruess et al., 2000) and Phillips (Phillips et al., 2011); (5) Intervention aimed at improving immune functions by behavioral therapy consisting of relaxation techniques, information about the “body-network”, health education, development and enhancement of stress- or illness-related coping skills (Schedlowski, 1994); (6) Mindfulness Based Stress Reduction (MBSR) consisting of guided awareness of bodily sensations, relaxation and breathing techniques, guided and mindfulness meditation practices, visualization and imagery techniques, gentle yoga (Matchim et al., 2011). For the study of Chan et al. (2006), we decided to take into consideration for meta-analysis only two intervention arms out of three due to having a structure and training manual backing the content of intervention: (7) Body-Mind-Spirit intervention (BMS) designed around four major themes related to normalization of traumatic experiences; “letting-go” of attachments; forgiveness and self-love and stabilization of long-term efforts to change through social support and commitment to help others. Further, (8) Supportive-Expressive (SE) subgroup designed around seven themes major part of which was dealing with fears and emotions and building relationships with family, friends. The third intervention [The Social Support Self-Help (SS)] of the study of Chan et al. (2006) was exempt because it did not include any structured program.

The studies measured both psychological variables and either different immune neuroendocrine parameters: lymphocytes (Schedlowski, 1994), NK Cells and PBMC Arginase (Baker et al., 2012), and cortisol or only cortisol (either salivary or plasma).

In all studies where salivary cortisol has been measured, several measurements have been made throughout the day. In the study of Hsiao et al. (2012), 6 daily measurements were performed (at wake-up time, 30 min after waking up, 45 min after waking up, at 12:00 h, at 17:00 h and at 21:00h); in the study of Chan et al. (2006), 5 measurements were taken (at wake-up time, 45 min after waking up, at 12:00 h, at 17:00 h and at 21:00 h); in the study of Baker et al. (2012), 3 measurements took place (at 8:00 h, at 14:00 h and at 20:00 h). Only in two studies 2 measurements of saliva were performed: in the study Dodds et al. (2015) (morning and afternoon) and in the study of Matchim et al. (2011) (at wake-up time and at 16:00 h).

In the studies where blood cortisol was measured (Schedlowski, 1994; Cruess et al., 2000; Phillips et al., 2011) the sample was withdrawn once per day at the same or nearly the same time: in the study of Cruess et al. (2000) blood was taken at 18:00 h, in the study of Phillips et al. (2011)- between 16:00 h and 18:30 h and in the study of Schedlowski (1994) - at 18:00 h (at baseline) and 20:00 h (post intervention).

For the studies with salivary cortisol, the log-transformed concentration, mean total level of daily (area under the curve, AUC) and the diurnal slope were examined. Cortisol (along with other parameters) was measured at baseline, after intervention and at follow up period. Duration of the intervention ranged from 8 weeks to 4 months, follow up reached up to 6 months (Baker et al., 2012) and 12 months (Phillips et al., 2011). All main characteristics of the studies are summarized in Table 1.

**Table 1:** Characteristics of included studies.

**Study**	**Design**	**Country**	**BC stage**	**Mean age** ±**SD**		**Sample**		**Intervention type**		**Outcome measures**	**Cortisol**	**Measurement timepoint**	**Time of cortisol measures**	**Interv, length**
				**Experimental group**	**Control group**	**Experimental group**	**Control group**	**Experimental group**	**Control group**					
Baker et al. (2012)	RCT pilot	UK	0-III	52.3 ± 11.3	53.3 ± 6.4	*n =* 6	*n =* 6	Haven psychological support program including complementary therapies for BC patients	Standard medical treatment alone	Psychosocial, immune, endocrine	Salivary	Baseline, 3 m, 6 m	AM, PM	4 m
Chan et al. (2006)	RCT	Hong Kong (China)	I-III	49.52 ± 6.94 (BMS) 46.88 ± 8.79 (SE) 50.3 ± 8.40 (SS)	47.47 ± 9.81	*n =* 27 (BMS) *n =* 16 (SE) *n =* 16 (SS)	*n =* 17	BMS (body mind spirit group), SE (supportive expressive therapy group), SS (social support self-help group)	Participants were given educational materials about nutrition, diet, body care after chemo-radiotherapy, edema	Psychological, physiological	Salivary	Baseline, 4 m, 8 m	AM, PM	8 m
Cruess et al. (2000)	Randomized waitlist control trial	Florida (US)	I-III	45.65 ± 7.61	45.65 ± 7.61	*n =* 24	*n =* 10	CBSM (cognitive behavioral stress management) therapy and relaxation techniques	Waitlist Controls	Cortisol, Benefit finding, Distress	Serum	Baseline, 10w	PM	10 w
Dodds et al. (2015)	Randomized waitlist control trial	Arizona (US)	I-IV	54.7 ± 12.1	55.8 ± 9.7	*n =* 12	*n =* 16	CBCT (cognitively based compassion training)	Waitlist Controls	Behavioral, psychosocial (self-report) and cortisol	Salivary	Baseline, 8 w, 12 w	AM, PM	8 w
Hsiao et al. (2012)	RCT	Hong Kong (China)	I-III	45.8 ± 6.9	46.5 ± 10.2	*n =* 26	*n =* 22	BMS (body mind spirit group) therapy	EDU (1xeducational session on health behaviors and emotional expression)	Beck depression inventory, meaning of life questionnaire, cortisol	Salivary	Baseline, 2 m, 5 m, 8 m	PM	2 m
Phillips et al. (2011)	RCT	Florida (US)	0-III	49.69 ± 7.89	49.69 ± 7.89	*n =* 63	*n =* 65	CBSM (cognitive behavioral stress management)	One day group-based psychoeducation seminar in lecture format on stress management techniques	Stress management skills (MOC2 questionnaire), cortisol	Serum	Baseline, 6 m, 12 m	PM	10 w
Schedlowski (1994)	Voluntary allocation control exploratory study, pre-post	Germany	I-III	51.0	50.6	*n =* 14	*n =* 10	Relaxation techniques, “body-network”, health education, guided imagery, enhancement of stress and illness related coping skills - twice a week	Controls received the same treatment two days after the second and 10th week intervention group.	Self-report coping questionnaire, lymphocytes, cortisol	Serum	Baseline, pre and post second session, pre and post 10th week	PM	10 w
Matchim et al. (2011)	Quasi experimental, pre-post control	Missouri(US)	0-II	56.87	61.47	*n =* 15	*n =* 17	MBSR (mindfulness based stress reduction) weekly teaching	No MBSR intervention	BP, heart rate. respiratory rate, mood disturbance, symptoms of stress and mindfulness state questionnaires, cortisol	Salivary	Baseline, following intervention and 1m after the program	AM, PM	8 w

### Data quantitative synthesis of salivary and plasma cortisol

**Figure 2:**
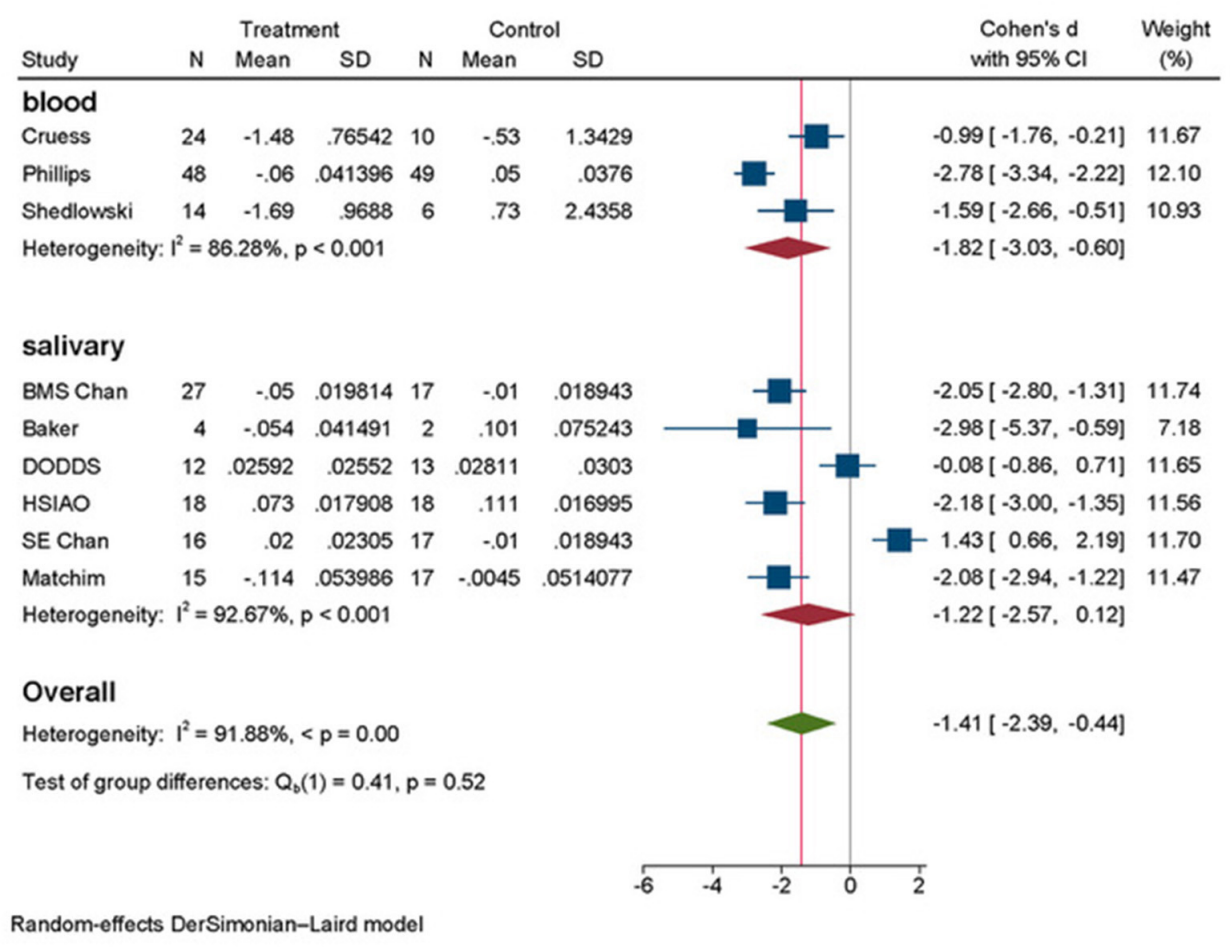
Forest plot subgroup analyses total by outcome measurements of cortisol.

**Figure 3:**
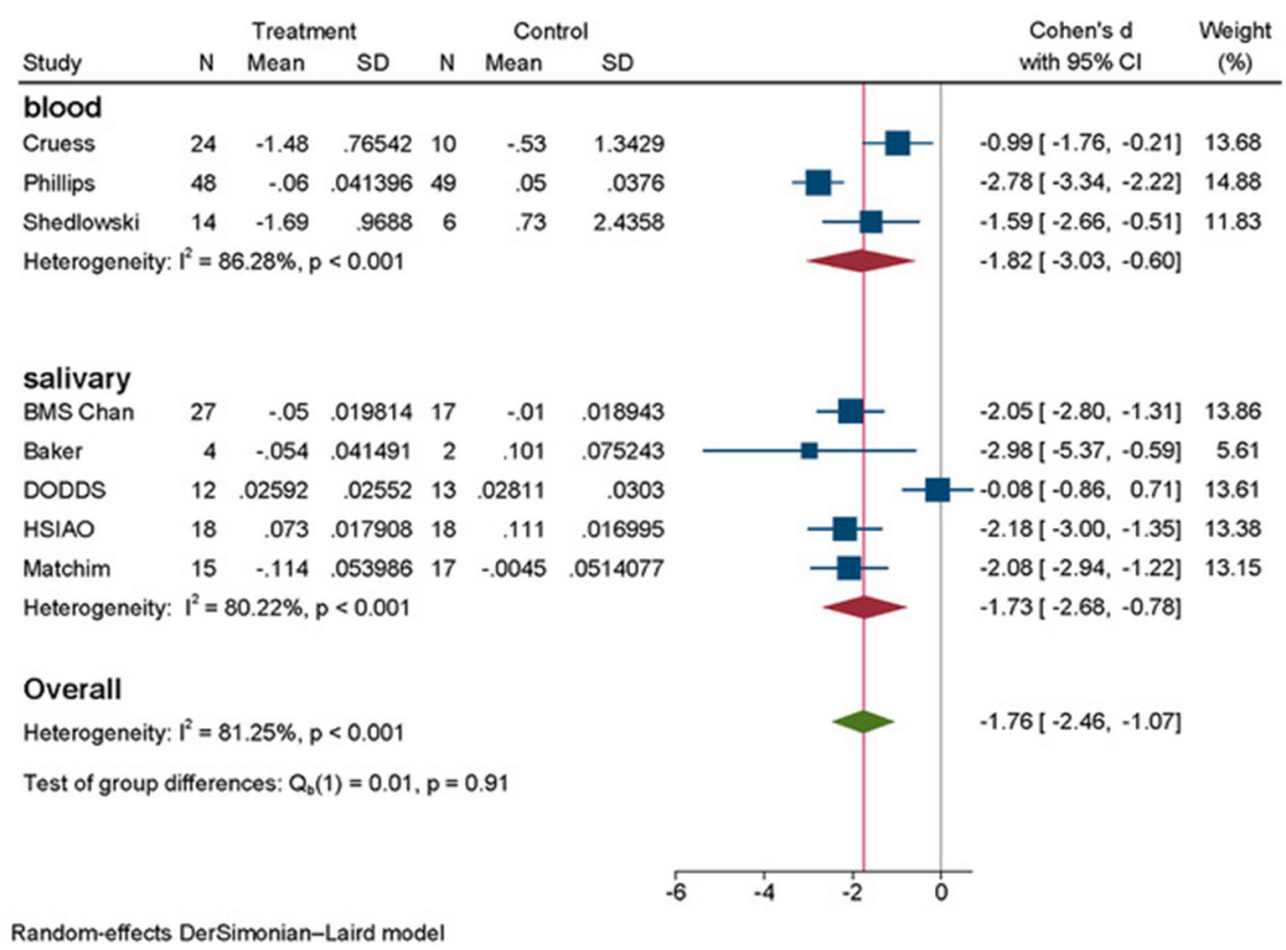
Forest plot subgroup analyses final by outcome measurements of cortisol.

We conducted random effects meta analyses where data could be pooled for eight studies, dividing them into two subgroups: six studies for salivary cortisol including both BMS and SE intervention arms of the study of Chan et al. (2006), and the studies of Matchim et al. (2011), Baker et al. (2012), Hsiao et al. (2012), Dodds et al. (2015) into one subgroup and the three studies for plasma cortisol (Schedlowski, 1994; Cruess et al., 2000; Phillips et al., 2011) into second subgroup. The results of meta-analyses are presented in Figures 2, 3.

Across the total studies examining association of psychosocial interventions on serum cortisol (Figure 2), we have found a Cohen's d = −1.82 (95% CI: −3.03, −0.60) as statistically significant. The forest plot for blood cortisol showed little visual evidence of heterogeneity, even though quantitative analysis presented high heterogeneity of I^2^ = 86.28%, *p* < 0.001. For the studies examining association of interventions on salivary cortisol, the effect size was not significant: d = −1.22 (95% CI: −2.57, 0.12) and heterogeneity of these studies examining salivary cortisol was high (I^2^ = 92.67%, *p* < 0.001), both traceable visually and quantitively. The intervention arm (SE) of the study of Chan et al. (2006) was falling to the right of the line 0 in forest plot and the study of Dodds et al. (2015) showed a very small effect size d = −0.08 (95% CI: −0.86, 0.71) seen on Figure 2.

Authors of Dodds et al. (2015) explained that their intervention consisted of presential classes and “at home” practice, where the adherence to was highly warranted. In the follow up analysis of the home practice, the severity and psychological distress scales of the fear of cancer recurrence demonstrated inverse correlation. All these factors might have accounted for the waning of the effect of overall intervention.

In the case of SE intervention arm of Chan et al. (2006) study, the intervention had adverse effect: d = 1.43 (95% CI: 0.66, 2.20). According to the authors, the SE group was perceived by participants as helpful, but no statistically significant changes were observed neither in psychological nor in physiological outcomes (cortisol). Again, the content of the intervention was largely dealing with the fears of death and dying and changes in the body image rather than positive reaffirmations. It was the prime reason for us to consider that this intervention arm did not fit into the semantic scope of other interventions that are subject of this research. Thus, certain psychological variables related to distress caused by fear of cancer recurrence, group small sample size, not equal environment of intervention and social factors might have influenced the outcomes adversely.

At this point we decided to remove the study of Chan et al. (2006) for SE Intervention arm to re-analyze the association of psychosocial interventions on salivary cortisol. In Figure 3 we present the results of this second meta-analysis. The effect size in salivary cortisol has increased to d = −1.73 (95%CI: −2.68, −0.78), and heterogeneity have decreased both visually and quantitively to I^2^ = 80.22%, *p* < 0.001.

Finally, the overall results in Figure 3 (combining psychosocial interventions that are similar yet different and combining salivary and plasma cortisol measurements), reached statistically significant very large effect size of psychosocial interventions on cortisol represented by d = −1.76 (95% CI: −2.46, −1.07) with heterogeneity analysis of I^2^ = 81.25%, *p* < 0.01. These results indicate the reduction of cortisol level due to applied psychosocial intervention as compared to control group or waiting list.

We have prioritized the result shown in Figure 3 as final for further discussion to confirm the hypothesis of effectiveness of psychosocial interventions for reducing chronic stress represented in cortisol measures in patients with BC, even though heterogeneity continues to be high (I^2^ = 81.25%, *p* < 0.001).

### Publication bias and sensitivity analysis

**Figure 4:**
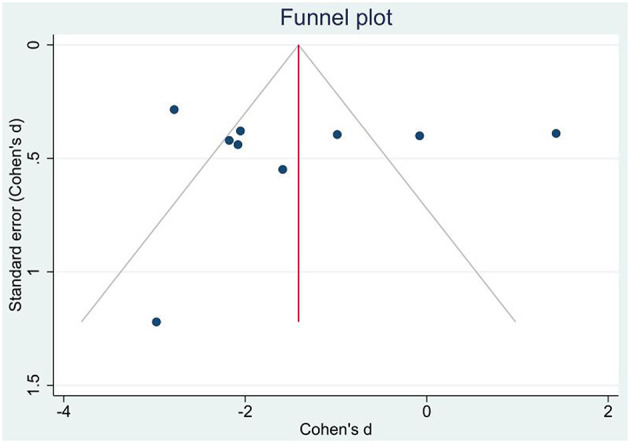
Publication bias funnel plot for standard errors and effect sizes of included studies.

We have tested the possibility of publication bias in the examined literature that was included in the above meta-analysis. For this purpose, we have drawn the funnel plot (Figure 4). Visual inspection of the funnel plot did not show asymmetry in the distribution of the studies, an indication that significant publication bias was unlikely. This was further confirmed by non-significant quantitative values of Egger's (*p* = 0.839) and Begg's (*p* = 0.754) tests.

### Risk of bias appraisal

**Figure 5:**
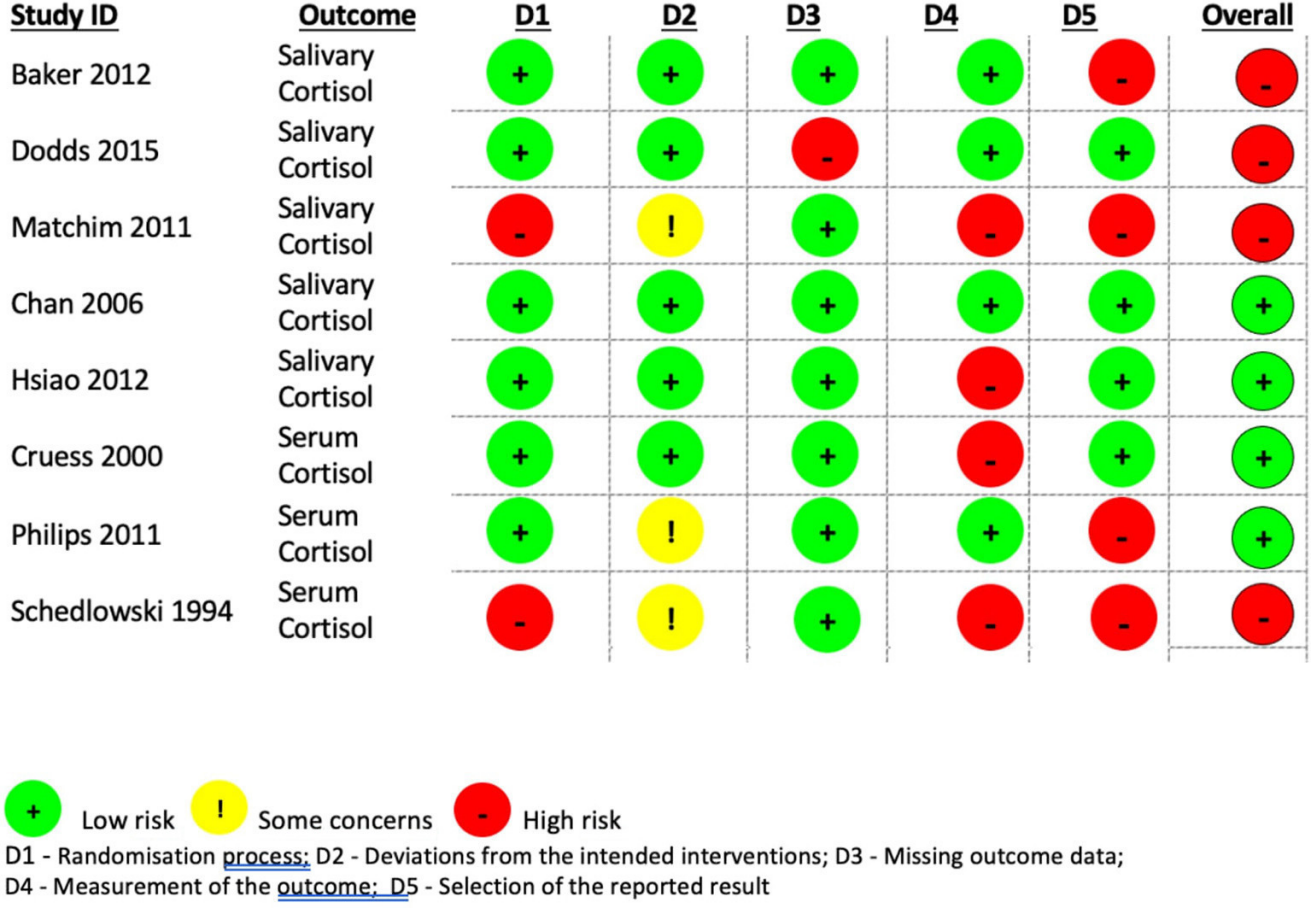
Risk of bias assessment of included studies.

The summary of risk of bias analysis is presented in Figure 5. The randomization process for most of the studies was respected. Only two studies (Schedlowski, 1994; Matchim et al., 2011) did not follow the randomization protocol by offering a voluntary participation either in intervention or control group due to ethical considerations. Otherwise, most of the studies present low risk of bias for domain 1. For the second domain, apart from the same two studies (Schedlowski, 1994; Matchim et al., 2011) where the participants were aware of the assigned intervention because of the voluntary participation, plus the study of Phillips et al. (2011) which provided no information on allocation concealment, present some concerns, whereas the rest of the studies are of low risk. For the missing outcome data domain all our studies present low risk since the outcome measures were well exhibited and the overall attrition was <20% (given rather small sample size of the studies and better traceability), except for the study of Dodds et al. (2015) where only 67% of randomized population received follow up. For domain four, most of the studies except four studies (Schedlowski, 1994; Cruess et al., 2000; Matchim et al., 2011; Hsiao et al., 2012) provided information that measurement of the outcome did not differ between groups, thus resulting in low risk. In the last domain, the quality of studies is decreasing; majority of the studies except four studies (Cruess et al., 2000; Chan et al., 2006; Hsiao et al., 2012; Dodds et al., 2015) present high risk due to reporting bias where there were multiple eligible analyses of the data. Finally, the overall risk of bias was determined to be balanced between low and high.

**Figure 6:**
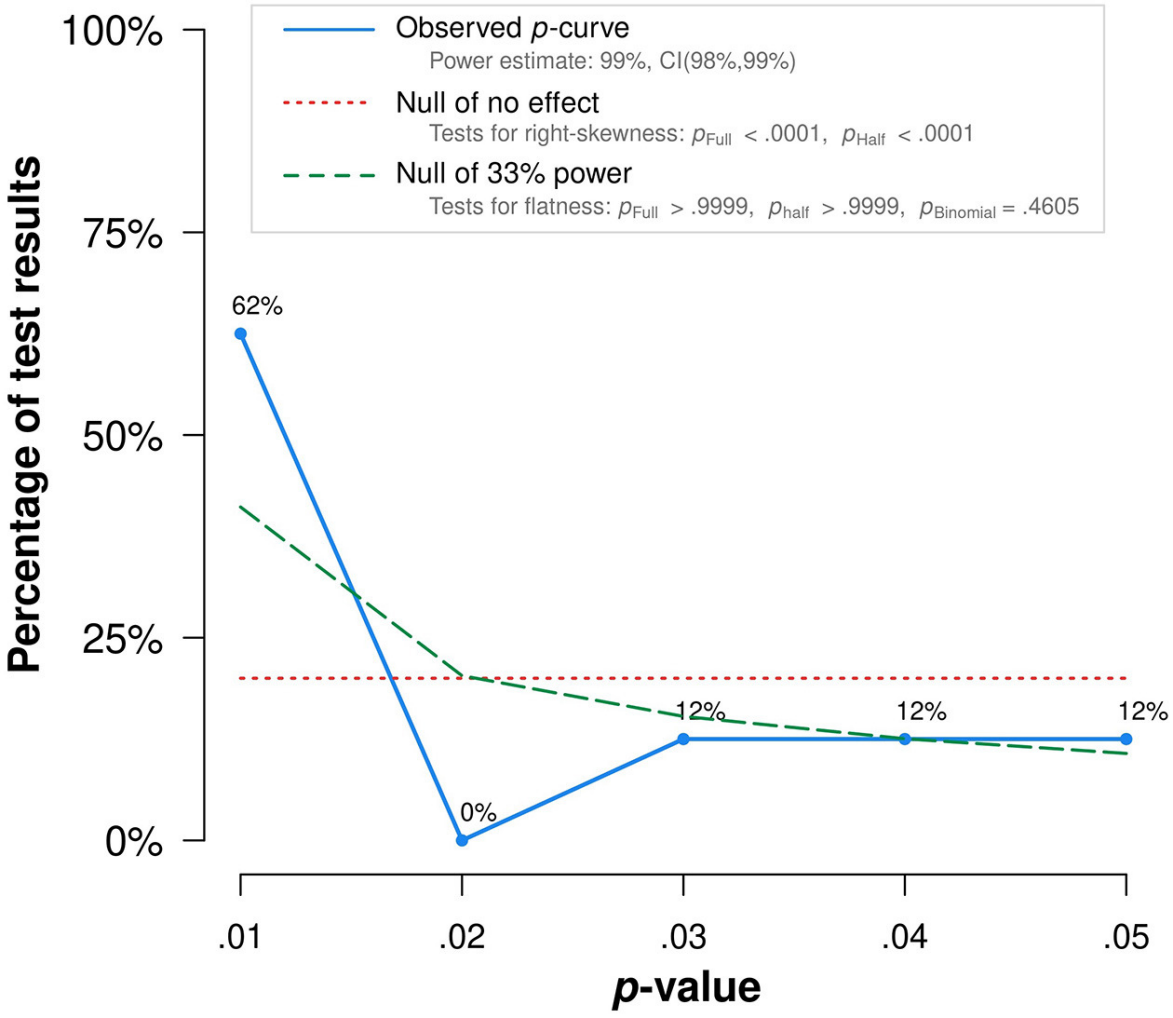
p-curve analysis.

We have performed a p-curve analysis to improve the risk of bias assessment. We have obtained a right-skewed p-curve which is diagnostic of evidential value (Figure 6).

## Discussion

The present meta-analysis aimed to test if the effect of certain types of psychosocial interventions on stress reduction can be traced by measuring cortisol in saliva or blood. We have based our research on a narrow niche of specific parameters by using cortisol as a biomarker to measure the effect size of specific psychosocial interventions (that are subject of this study) on chronic stress reduction.

Psychosocial interventions in mental health use non-pharmaceutical means to alter a person's behaviors and relationships with society, to reduce the impact of the person's disorder or condition and manage the causal issues better. Interventions associated with psychosocial support fall under two main umbrellas of therapy: cognitive therapy and behavioral therapy, or collectively - cognitive behavioral therapy and its sub-categories.

In our research we determined that the applied interventions within these two major arms providing non-invasive care had overall impact on the BC patients' level of cortisol. The scope of these interventions included: Mindfulness Based Stress Reduction, Cognitive Behavioral Stress Management, Mind Body Spirit integrated therapy, cognitive restructuring, cognitive based compassion therapy, hypnotic guided imagery program, coping skills development, psychoeducation, supportive expressive therapy, behavioral modification program, spiritual and psychosocial support, various relaxation techniques, biofeedback, complementary medical therapies involving movement in meditation modalities. All these programs emphasized positive aspects and emotionally uplifting assertions or techniques. In the studies measuring salivary cortisol (Matchim et al., 2011; Baker et al., 2012; Hsiao et al., 2012) and BMS Intervention arm of Chan et al. (2006) study the applied interventions decreased the level of cortisol in patients. In the studies of Schedlowski (1994), Cruess et al. (2000), and Phillips et al. (2011) using plasma cortisol measurements, the reduction of cortisol due to intervention was even more noticeable, thus providing stronger evidence of this relationship. These results confirmed our hypothesis that psychosocial support and mental adjustment can decrease stress marker (cortisol) in patients with BC. However, as described in Results section, interventions [study of Dodds et al. (2015) and SE intervention arm of the study of Chan et al. (2006)] that dealt partially or entirely with negativity aspects like fears of recurrence, death, dying, or accentuating distress had no effect or had adverse effect on the level of cortisol in patients with BC.

Studies of Schedlowski (1994), Cruess et al. (2000), and Matchim et al. (2011) provided information that cortisol level can be reduced rather at the intervention completion, thus showing short-term effect. Yet the study of Baker et al. (2012) registered positive results of intervention at 6 months follow up, whereas the studies of Chan et al. (2006), Phillips et al. (2011), Hsiao et al. (2012) have presented that CBSM and BMS interventions strongly predict decreased cortisol levels up to 8 or 12-months follow up respectively. The evidence that intervention is effective in applying stress reducing and relaxation habits after 6 months and plateauing afterwards suggests that there might be a need for maintenance sessions.

According to our knowledge no other meta-analysis has dealt with this specific and comparable biomarker of cortisol for a specific target population and grouping certain types of psychosocial interventions that dealt with determined focus of positive assertion, hope, encouragement, and self-regulation techniques (as opposed to emphasizing fears). The study's limitations conclude small sample size of each study, not following randomization principle in some studies, high risk of bias, some heterogeneity of the groups and quality of support in interventions. Due to small sample size, the source of heterogeneity through sensitivity analysis, meta regression and subgroup analysis was not possible to perform. This implies the necessity of further investigation in this field to be able to provide a solid base for the preventive care. Finally, to optimize patient care through understanding the relationship between the influence of glucocorticoid signaling on cancer biology to be able to implement adequate psychosocial interventions and revert the disease progression will require stronger coordinated efforts between oncologists and psychology researchers.

Our research is supported by previous similar findings that cannot be ignored falling into three main pillars of scientific evidence.

Firstly, exposure to physiological stress has been proposed to increase cancer risk and recurrence (van der Pompe et al., 1997, 2001), however combined effects of stress accompanying psychological depression and disruption of normal social life that seriously impair BC females' quality of life and wellbeing, when demonstrated, have been inconclusive. Certain personality types may enhance or degrade immune response (Oswald et al., 2006). Chronic stress has been associated with suppression of immune function and elevations in inflammatory activity, and there is evidence that the immune system may not adapt over time, while cortisol's immunosuppressive effect might be involved in cancer development, causing further oncogenic mutations (Thornton et al., 2009). Some studies have revealed that abnormal diurnal cortisol rhythms are related to poor prognoses in BC patients (Spiegel et al., 1998; Sephton et al., 2000). Thus, stress, playing a mediating role in immune modulation, might be involved in cancer development (including recurrence), while psychosocial interventions may conversely reduce stress induced suppression of immunity, hence improving cancer survival by buffering the biological effects of stress (Fife et al., 1996).

Secondly, cortisol is included within inflammatory and immune processes, acting as an anti-inflammatory agent by causing apoptosis through the mitochondrial pathways. It has been extensively used for measuring HPA axis functioning and body's immunity, and can equally demonstrate the link of psychological stimulation, emotions, and physiological changes (both hormonal and immunological). The study of Matousek et al. (2010) indicates, that in conjunction with other biological measurements, the use of cortisol levels as a physiological marker of stress may be useful to validate even self-reported results of attributed intervention. Molecular studies demonstrate a clear overlap between intracellular stress signaling and protumorigenic pathways within breast cells but need to be integrated with other stress-BC research in order to obtain an unambiguous assessment of the potential cause-effect relationship (Antonova et al., 2011). The findings regarding specific pathways of molecular mechanisms and increased glucocorticoid signaling on the immune system and systemic metabolism related to cancer progression are described elsewhere (Volden and Conzen, 2013).

Comprehensive study of cortisol biomarker's variability due to stress modulating psychological interventions might be used as a novel prognostic factor for disease development (progression, recurrence, or recovery). Cortisol is a reliable tool to assess the adrenocortical response, yet easy and non-invasive means to perform a repeated and “non-disturbing” sampling. Salivary cortisol represents the biologically active form of the hormone, and a strong association between levels in blood and saliva has been described in animal studies. Peeters et al. (2011) indicated that total blood cortisol concentrations might account for 80% of salivary cortisol concentrations and vice versa.

A strong basal diurnal rhythm of cortisol exists: it has a tendency to surge in the morning upon waking up, increase 50–60% in the first 30–45 min after awakening, drop rapidly over the first few hours after waking, and then decline more slowly across the day to reach a low point around midnight. Therefore, it requires certain methodological prerequisites in sampling process to avoid confusion factors (circadian variations, age, gender, smoking) (Weibel, 2003). As seen in the results of this study, the temporal variability of salivary cortisol is higher than that in the plasma cortisol added to the overall diurnal tendency of fluctuation, and it is more complicated to assess the accuracy of the measurements. In our research most of the included studies followed rigorous methods in collecting salivary or plasma cortisol (using high sensitivity cortisol enzyme immunoassay or enzyme-linked immune-absorbent assay kit or intra-interassay sensitivity kit) and providing demographic overview of the participants. Also, to control cortisol circadian rhythm in the reviewed studies, several measures have been taken. In our meta-analysis, we have considered the mean cortisol value throughout the day. In this way we tried to control for diurnal changes in cortisol levels. In the studies where blood cortisol was measured, only one cortisol measurement was collected as this procedure is more invasive than the saliva sample. However, in all of them the blood was taken at the same or nearly the same time of the day. This also decreases the effect of variability in blood cortisol levels throughout the day.

Thirdly, past research in PNI has been investigating various sides of this large topic: there are some extensive narrative reviews dedicated to the subject of PNI in general (Mulder et al., 1992; Bauer, 1994; Garssen and Goodkin, 1999; Schleifer, 2007; Wahbeh et al., 2009; Subnis et al., 2014; Jassim et al., 2015; Hulett and Armer, 2016). Further, the effect of psychological and psycho-behavioral interventions on cancer in general and on BC patient's immune function (Tong et al., 2014; Zhang et al., 2016, 2019; Guarino et al., 2020). Finally, the impact of psychosocial interventions on survival time in cancer patients (Chow et al., 2004; Smedslund and Ringdal, 2004; Oh et al., 2016). Some of these published meta-analyses used specific neuroendocrine biomarkers as outcome variable and as predictors for recurrence of BC (Sephton et al., 2000; Tong et al., 2014; Zhang et al., 2016, 2019).

To understand the ambiguity of this large and complex topic, we have further examined that several previous reviews show little or no evidence that psychosocial interventions improve survival time (Jassim et al., 2015) or demonstrate only short-term improvement in survival (Chow et al., 2004). Although there is a considerable evidence of psycho-behavioral interventions' effect on immune indicators, the effect size of meta-analysis of Tong et al. (2014) is modest and the authors highlighted that intervention focus and comparable immune measures should be taken into consideration for future research. Due to heterogeneity in various studied aspects, meta-analysis in some of these reviews was not possible (Mulder et al., 1992; Hulett and Armer, 2016).

Even though the results of the reviews of Tong et al. (2014), Hulett and Armer (2016), and Zhang et al. (2016) are inconclusive, the authors suggest that interventions with mindfulness-breathing-stretching components, cognitive–behavioral therapies or relaxation training appear to offer potential improvement or stabilization of neuroendocrine-immune activity through PNI interactions in patients with cancer compared to control groups. There is growing evidence that psychosocial interventions improve the wellbeing of cancer patients, producing favorable effects on some psychological outcomes, such as anxiety, depression, and mood disturbance. Most of the studies included in the above meta-analyses suffered from small sample size and poor compliance, especially in studies involving patients with advanced disease. Although psychosocial interventions appear to be promising adaptation therapy against many of the psychophysiological impacts of cancer, according to the review of Smedslund and Ringdal (2004) they should be thoroughly defined, focus on a single diagnosis, and consider known risk factors.

If chronic psychological distress is associated with health-relevant behavioral and biological processes in cancer patients, then it is plausible that certain types of psychosocial interventions aimed to reduce distress and promote psychological adaptation could possibly influence health outcomes particularly in BC patients (McGregor and Antoni, 2009). The effect of psychological factors has been more related to cancer progression, recurrence and metastasis rather than cancer initiation (Mulder et al., 1992). The preliminary evidence of mindfulness based, or similar psychosocial supportive care, more convincingly in interventions, shows the reduction of pro-inflammatory cytokine production response in cancer patients that may strengthen coping capacity and improve BC survivorship. Relating stress biology and pro-metastatic molecular processes, clinical researchers are now looking to pioneer further development in research to mitigate adversity of cancer recurrence in part by down-regulating similar pathways (Lutgendorf et al., 2010) and link effects of chronic stress on BC progression (Sloan et al., 2010).

Even though it is still premature to conclude the effectiveness of these interventions among cancer patients, there is a call to acquire evidence of association between interventions that improve emotional wellbeing, quality of life, and objective indicators like survival /mortality/relapse/recurrence and immune responses. The evidence for efficacy of psychosocial interventions in cancer care is unequivocal, however, there is a need for an objective marker of improvement in research evaluating specific psychosocial programs standardizing content, duration, frequency, follow up and measurement methodology. Additional caveats should include analysis of the type, focus and quality of patient's support network and more personalized treatment plan. Further studies of high quality and large sample sizes are required for more solidified conclusions. We continue to explore this domain of PNI of cancer where immune reactions are more sensitive and responsive (yet measurable) to psychological influences aimed at improving overall health outcome. Results of this meta-analysis could be a promising steppingstone to grant evidence-based foundation in routine medical care.

## Conclusion

Our study has concluded that certain types of psychosocial interventions reduce cortisol (indicator of chronic stress) in patients with BC. Application of specific psychosocial support as adjuvant non-invasive therapy for affected females with BC at all phases of treatment could contribute to more cost-effective health care.

## Data availability statement

The original contributions presented in the study are included in the article/supplementary material, further inquiries can be directed to the corresponding authors.

## Author contributions

EM, NG-C, and RL-G contributed to conception and design of the study and wrote the first draft of the manuscript. EM-L and MA-S wrote sections of the manuscript. AM organized the database. MG-B performed the statistical analysis. All authors contributed to manuscript revision, read, and approved the submitted version.
